# Exploring Cervical Adenocarcinoma: Epidemiological Insights, Diagnostic and Therapeutic Challenges, and Pathogenetic Mechanisms

**DOI:** 10.1002/cam4.70620

**Published:** 2025-01-22

**Authors:** Shuhui Li, Congrong Liu, Liang Weng

**Affiliations:** ^1^ Department of Pathology, School of Basic Medical Sciences Peking University Health Science Center Beijing China; ^2^ Department of Pathology Third Hospital, Peking University Health Science Center Beijing China

**Keywords:** cervical adenocarcinoma, cervical cancer, gynecological oncology, organoid, single‐cell RNA sequencing, tumor microenvironment

## Abstract

**Background:**

Cervical cancer poses a significant threat to women's health and encompasses various histological types, including squamous cell carcinoma (SCC), cervical adenocarcinoma (CA), and adenosquamous carcinoma. CA, in particular, presents a formidable challenge in clinical management due to its low early detection rate, pronounced aggressiveness, high recurrence rate, and mortality, compounded by the complexities associated with late‐stage treatment. There is limited understanding of the similarities and differences in the pathogenesis mechanisms between CA and SCC, such as tumor heterogeneity and the tumor immune microenvironment (TME).

**Methods:**

A literature search was carried out in the PubMed, Web of Science, and Google Scholar databases using the following research terms: “gynecological oncology,” “cervical cancer,” “cervical adenocarcinoma,” “epidemiology,” “diagnosis and treatment of cervical adenocarcinoma,” “Human papillomavirus,” “World Health Organization,” “tumor microenvironment,” “single‐cell RNA sequencing,” “molecular mechanism,” and “preclinical research model.”

**Conclusion:**

This review consolidates the epidemiological characteristics, diagnostic and therapeutic hurdles, and the latest advances in research on CA. It aims to highlight the significant heterogeneity of the TME characteristics exhibited by CA compared to SCC. Additionally, we also summarize the common preclinical models for CA and discuss the advantages and disadvantages of using various models in research. We aspire that the discussions presented herein will offer novel insights and directions for subsequent research, as well as clinical diagnosis and treatment strategies for CA.

**Abbreviations**
CA: cervical adenocarcinoma
CAFs: cancer‐associated fibroblasts
CT: computer tomography
HPV: human papillomavirus
MDA: minimal deviation adenocarcinoma of cervix
MRI: magnetic resonance imaging
MSI: microsatellite instability
PD‐L1: programmed cell death 1 ligand 1
PDX: patient‐derived tumor xenograft
SCC: squamous cell carcinoma
ScRNA‐seq: single‐cell RNA sequencing
SLC26A3: solute carrier family 26 member 3
SnRNA‐seq: single nuclei RNA sequencing
TCR‐seq: T‐cell receptor sequencing
TMB: tumor mutational burden
TME: tumor immune microenvironment
Tregs: regulatory T cell
WHO: World Health Organization

## 1 Introduction

Cervical cancer constitutes 6.24% of all cancer cases among women, comprising squamous cell carcinoma (SCC), cervical adenocarcinoma (CA), and other epithelial tumors [1]. SCC is the most common type, accounting for about 80%, while CA makes up 10%–25%. Despite a declining incidence of cervical cancer overall, the incidence of CA is rising compared to SCC [2]. Persistent infection with high‐risk human papillomavirus (HPV) types, particularly HPV16, 18, and 45, is the primary cause of CA, responsible for about 75% of cases. However, HPV infection is undetected in about 15% of CA cases [3]. Standard diagnostic methods for cervical cancer, such as cervical cytology and HPV testing, have relatively low detection rates for CA, leading to potential oversight or misdiagnosis. Additionally, CA poses risks of recurrence and metastasis following treatment, with poorer prognosis [2, 4]. Therefore, early screening, diagnosis, and treatment of CA remain urgent challenges that needs to be addressed.

Tumor immune microenvironment (TME) is a complex ecosystem comprising of blood vessels, immune cells, stromal cells, signaling molecules, and the extracellular matrix. Paget's "seed and soil" hypothesis highlights the tumor‐TME interaction's role in tumorigenesis [5]. The pathogenesis of CA exhibits a close interplay with the TME. Specifically, the tumor can subtly exploit its microenvironment by enhancing angiogenesis to promote its growth and dissemination, while simultaneously suppressing the immune response of the organism [6]. Omics technologies and single‐cell RNA sequencing (scRNA‐seq) have enabled precise identification of TME cell subpopulations and genomic mutations, revealing tumor growth, evolution, and molecular mechanisms. Numerous studies have been conducted on the TME of SCC, yet there are only a handful of reports on the microenvironment of CA. This article summarizes CA knowledge, offering new insights and strategies for diagnosis, treatment, and prevention.

## 2 Epidemiology of CA

According to the latest global cancer statistics, approximately 604,127 new cases of cervical cancer and 341,831 deaths were recorded in 2020. The incidence of cervical cancer varied globally, declining with the increase in the Human Development Index. The incidence of SCC is higher in developing countries, while CA is on the rise in some high‐income countries [7]. Globally, over 58% of cervical cancer cases are estimated in Asia, followed by Africa (20%), Europe (10%), and Latin America (10%). Similarly, more than half of cervical cancer deaths occur in Asia (58%), followed by Africa (22%) and Latin America (9%). China and India account for 39% of cases (18% and 21%, respectively) and 40% of deaths (17% and 23%, respectively) [8, 9]. In China, gynecological cancer cases rose from 177,839 to 241,800 from 2007 to 2016, with cervical cancer growing fastest at 4.1% [10]. HPV 16 and 18 are the most common cervical cancer subtypes, accounting for 70% of cases. Other risk factors include high parity, smoking, multiple sexual partners, and HIV infection. Globally, 4.9% of new cervical cancer cases are linked to HIV [11]. In cervical cancer, although the incidence of CA is relatively lower compared to SCC, CA may exhibit a higher degree of malignancy, lower survival rates, and a greater risk of distant metastasis [12, 13]. The decline in the incidence of cervical cancer was primarily attributed to the reduction in SCC, whereas the increasing proportion of CA might be a significant factor contributing to the stagnation in survival rates.

## 3 Diagnosis and Treatment of CA

The 2020 World Health Organization (WHO) classification redefined CA histological types as HPV‐associated and HPV‐independent (Table 1). Unlike almost 100% HPV‐associated SCC, 15%–20% of CA is HPV‐independent, including gastric, mesonephric, and clear cell types [14, 15]. This update offers new insights into CA pathogenesis, prevention, and treatment.

**TABLE 1:** WHO classification of female genital tumors. 5th edition.

WHO classification of female genital tumors. 5th edition	
HPV‐associated CA	HPV‐independent CA
Usual type (~75%)^a^ Mucinous type (~10%)^b^ ○ Mucinous NOS adenocarcinoma ○ Intestinal adenocarcinoma ○ Signet‐ring cell adenocarcinoma ○ Stratified mucin‐producing carcinoma Adenocarcinoma, HPV‐ associated, NOS	Gastric type (10% ~ 15%) Clear cell type (3% ~ 4%) Mesonephric type (< 1%) Adenocarcinoma, HPV‐independent, NOS

^a^ Usual type: Cells with mucinous cytoplasm constitute only 0%–50% of the tumor.

^b^ Mucinous type: There is intracytoplasmic mucin in 50% of cells, typically with a minor component of usual adenocarcinoma.

**FIGURE 1:**
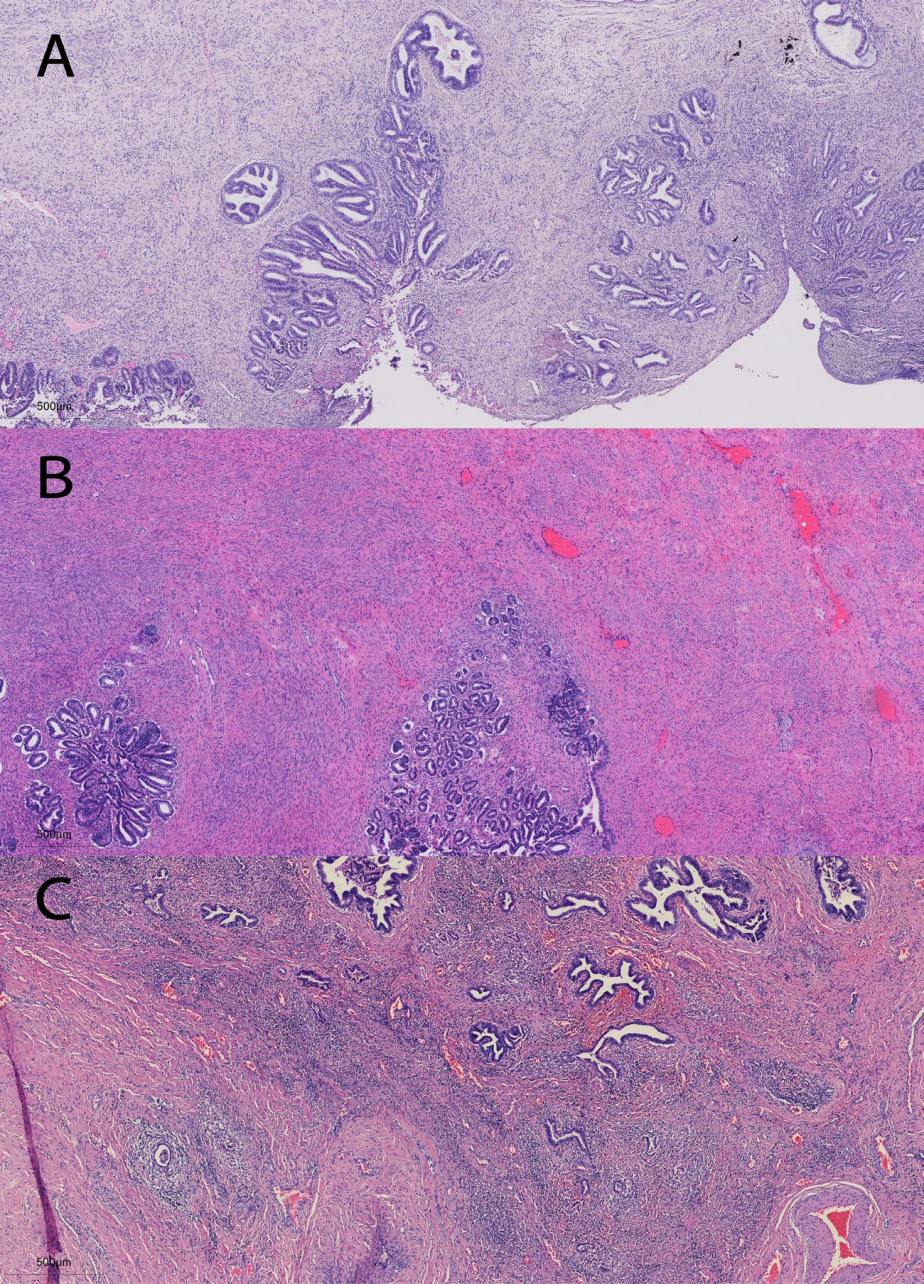
The representative HE‐stained pathological images are classified according to the Silva system (A–C), with an original magnification of ×4. (A) Silva A tumor glands are rounded and distributed in clusters. (B) Silva B tumor with most of the glands still growing as Silva A, with focal appearances of infiltrating mesenchyme. (C) Silva C tumor is open glandular with diffuse mesenchymal destructive infiltration.

The diagnosis and treatment of cervical cancer primarily rely on cervical cytology and HPV testing, complemented by gynecological examinations, imaging studies, pathological assessments, and tumor marker screening [16, 17]. The squamous carcinoma‐associated antigen is a significant marker for SCC of the cervix, and carcinoembryonic antigen, carbohydrate antigen CA‐125, and CA‐19‐9 can also be elevated [18, 19]. A histological biopsy, performed under colposcopy or direct visualization, serves as the gold standard for confirming cervical cancer. In cases where biopsy results are negative but cancer cannot be clinically ruled out, cervical cone biopsy combined with pathological examination emerges as an effective diagnostic approach [20]. Due to variations in the interpretation of cervical cancer cells among pathologists, the Silva team introduced a novel classification system for the usual type of HPV‐associated CA. This method emphasizes biological behavior, enabling more accurate prognostic predictions and guiding treatment decisions [21]. It offers a new perspective on the diagnosis and treatment of cervical adenocarcinoma (Table 2, Figure 1). Imaging modalities, including magnetic resonance imaging (MRI) and computed tomography (CT), assess the extent of tumor invasion, lymph node status, and distant metastases in advanced stages, providing crucial information for a comprehensive medical history evaluation [22]. Upon pathological confirmation of cervical cancer through biopsy, the clinical team devises a surgical plan that incorporates imaging data. Surgical interventions or surgery‐centric approaches dominate the treatment of early‐stage cervical cancer, while platinum‐based chemoradiation therapy is preferred for advanced stages [23].

**TABLE 2:** Silva score criteria and clinical significance of CA.

Silva classification	Growth pattern of neoplastic glands	Mesenchyme	Prognostic predictive value and treatment modalities
A	(1) The glands are round, complete in outer contour, and distributed in clusters. (2) There are no individual free tumor cells and a solid growth pattern is lacking. (3) The glands may have a complex growth pattern such as sieve and papillae within the glands.	(1) No destructive interstitial infiltration. (2) No vascular invasion.	No metastasis, no recurrence A clean margin for taper cuts is sufficient
B	(1) Single tiny clusters of tumor cells were separated from rounded glands, with single, numerous, or linearly ordered tumor cells seen at the tumor's base. (2) Lack of a solid growth pattern.	(1) Intact contoured glandular foci generate a damaging interstitial infiltration, with fibrotic tissue hyperplasia and inflammation in the surrounding interstitial foci. (2) Vascular invasion can be seen.	Occasional metastasis, rare recurrence Positive sentinel lymph node biopsy: radical hysterectomy + lymph node dissection
C	(1) Angular glands or small lumen interspersed with open glands. (2) Within a 5 mm field of view, glands, papillae, and mucous lakes exhibit a fused growth pattern. (3) A distinct solid growth pattern is visible.	(1) Diffuse destructive infiltration is characterized by a widespread infiltration of glands accompanied by a profound proliferative reaction of fibrous connective tissue, indicating a significant fibrotic response. (2) Vascular invasion can be seen.	Often metastatic, often recurrent Radical hysterectomy + lymph node dissection with adjuvant therapy if necessary

CA poses significant challenges in both diagnosis and treatment. HPV‐associated and HPV‐independent cases require different diagnostic approaches. For patients testing positive for HPV types 16 and 18, direct referral to colposcopy for histological biopsy is recommended to ascertain lesion characteristics [24]. When HPV test results are positive, the actual diagnostic accuracy for CA ranges from 70% to 90%, while the misdiagnosis rate may vary between 10% and 30% [25]. Conversely, HPV‐independent patients necessitate a combination of tests for proper diagnosis. Screening for HPV‐independent CA may not occur in a timely manner, leading to delayed diagnoses and worsened prognoses [26]. CA is more challenging to detect early through routine screening methods compared to SCC. The small and dispersed nature of CA lesions further increases the risk of missed or misdiagnosis [27, 28]. Therefore, diagnosing CA requires careful consideration of various factors, including clinical manifestations, gynecological examinations, cytology, colposcopy, imaging studies, and histopathological assessments.

In terms of treatment, CA exhibits higher rates of recurrence and metastasis compared to SCC, and current therapeutic approaches have limitations in controlling these processes. Patients with early‐stage CA may achieve a better prognosis through surgical interventions, such as radical hysterectomy. However, for patients with advanced‐stage CA, the severity of the disease significantly complicates treatment and reduces efficacy. For stage I CA, the 5‐year survival rate is generally high, typically ranging from 70% to 90%. However, when the disease progresses to stages II and III, the 5‐year survival rate significantly decreases, usually falling between 30% and 60%. In cases of recurrent disease, the success rate of treatment is often low, depending on the chosen treatment plan and individual patient responses [2, 29]. Bevacizumab combined with paclitaxel and cisplatin is a standardized treatment regimen for cervical cancer, showing good efficacy in SCC but less so in radio‐ and chemo‐resistant CA [30].

To summarize, identifying and treating CA remains difficult, necessitating a more thorough and complete examination and more effective biomarkers to enhance patient outcomes and prognoses.

## 4 TME in CA

### 4.1 Overview and Characteristics of TME

**FIGURE 2:**
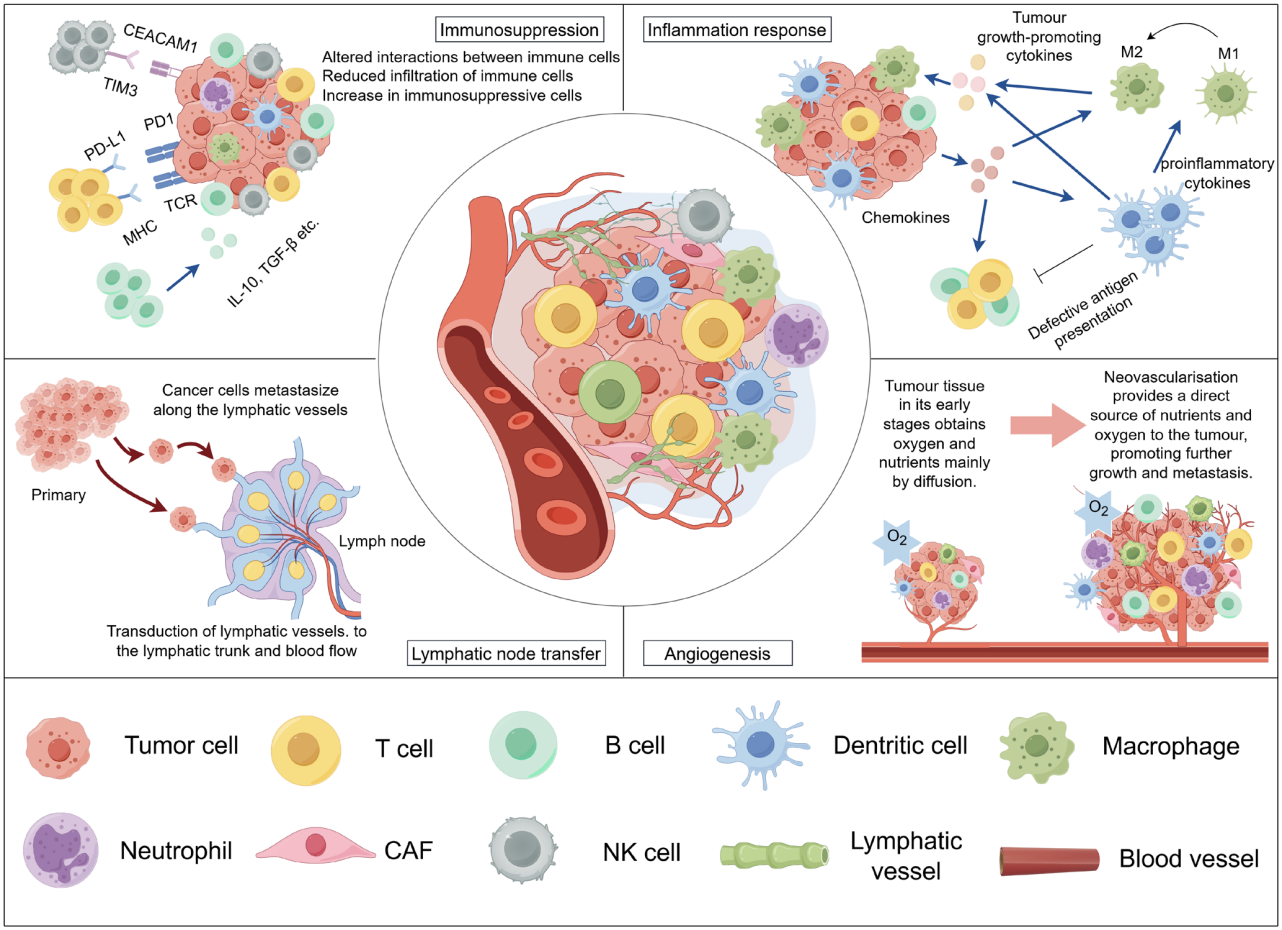
Key features of TME.

Cancer evolves from normal to malignant cells by overcoming barriers. During the progression of cancer, TME, comprising immune and stromal cells, plays a crucial role [31, 32, 33] (Figure 2). These complex interactions make cancer treatment challenging, requiring multifaceted consideration.

The immune system initially defends cancer, but cancer cells evade surveillance, altering the TME. The inflammatory response can either promote the proliferation, invasion, and metastasis of tumor cells or inhibit the development of tumors by activating immune cells. Angiogenesis and lymphatic node transfer fuel cancer growth and dissemination.

## 5 Research on the Mechanism of TME in CA

The study revealed significant differences between SCC and AC in terms of both genetic mutations and TME. Among SCC patients, the genes with the highest mutation frequencies are *PIK3CA* (46.9%), *FBXW7* (18.1%), *TERT* (15.7%), *EP300* (15.4%), and *LRP1B* (12.2%). In contrast, forCA patients, the genes with the highest mutation rates include *TP53* (42.7%), *PIK3CA* (32.9%), *KRAS* (23.2%), *ERBB2* (19.5%), *ARID1A* (17.1%), *PTEN* (14.6%), *STK11* (14.6%), *SMAD4* (12.2%), and *CDKN2A* (11%). Notably, genetic alterations in AC, such as *KRAS*, *ERBB2*, and *ARID1A*, are significantly associated with a decrease in B cell, T cell, and dendritic cell infiltration, while the *EP300* mutation in SCC is associated with an increase in CD4(+) T cell infiltration [34]. Additionally, Punt et al. [35] pointed out that the distribution and function of regulatory T cells (Tregs), IL‐17(+) cells, and Th17 cells in CA differ from those in SCC. Specifically, Tregs and IL‐17(+) cells appeared much more frequently in the tumor stroma than in the tumor epithelium, and only a small number of Th17 cells were observed in CA. Of these, Tregs and IL‐17(+) cells represent a beneficial immune response, and Th17 cells may represent an adverse response to CA.

Recent omics advances, especially scRNA‐seq, let researchers identify cell subpopulations, analyze TME, and characterize cellular mutations. This offers new insights into CA pathogenesis [36] (Table 3).

**TABLE 3:** Function of the TME in different cervical cancers.

Cancer		Cell type	Cluster	Function	Cite
SCC		Malignant epithelial cell	×	*KRT6A*, *TP63*, *CDKN2A*, *KRT13*, *DSG3 ↑* Immune‐related pathways *↑*	[37]
				Cytokine interaction *↑* ECM interaction *↑*	[38]
				*S100A8*, *KRT14 ↑*	[39]
		T cell	CD8+ T	Immunological activity status, toxicity, proliferation, exhaustion, and regulation *↑*	[37]
			CD8+ T	Cytotoxic *↑* Regulatory *↑*	[38]
		Macrophage	×	Antigen‐presenting genes and *S100A* family *↑*	[37]
			MRC1 + Macrophage	Phagocytosis *↑*	[38]
		CAFs	×	Have similar characteristics to tumor cells and are involved in immune regulation	[37]
			vCAFs, myoCAFs	Migration *↑*	[38]
			iCAF, my CAFs	Tumor progression ↑	[39]
CA	HPV‐associated	Malignant epithelial cell	×	*EPCAM*, *MUC5B*, *KRT8*, *KRT18*, *AQP3 ↑* Pathways related to lipid metabolism and triglyceride metabolism *↑*	[37]
				Mismatch repair *↑* N glycan biosynthesis *↑*	[38]
				*CDKN2A*, *CLDN3、CLDN4*, *GDF15 ↑*	[39]
			Epi_09_SST, Epi_10_CYSTM1	*SLC26A3*, *ORM1*, *ORM2 ↑* Pluripotency and differentiation potential	[40]
		T cell	CD8 + T, CD4 + T	Immunization ↓	[37]
			T_ex	Exhausted *↑*	[38]
			Tregs	Immunosuppression	[40]
		Macrophage	×	Associated with high expression of cytokines, chemokines, and proliferation‐related genes	[37]
			×	Immune Regulation *↑*	[38]
		CAFs	×	Similar to normal fibroblasts, play an important role in tissue remodeling	[37]
			dCAFs	Development *↑*	[38]
			iCAF, my CAFs	Tumor progression ↑	[39]
	HPV‐independent	Malignant epithelial cell	×	High developmental diversity and poor differentiation	[39]
			Epi_12_RRAD	*ADH1C*, *IGF2*↑	[40]
		T cell	Tregs	Immunosuppression	[40]
			Th17 cells, CD4 + cytotoxic cells, exhausted CD8 + T	Immune system dysfunction and tumor development	[41]
		CAFs	iCAF, my CAFs	Tumor progression ↑	[39]
			×	Promote the proliferation of tumor cells and stromal cells and potentially be able to recruit immune cells into the tumor stage	[41]
		Dendritic cell	Mature Dendritic cell	Recruited Th17 cells into the tumor region via cytokines	[41]

Li et al. [37] analyzed SCC and CA tumor ecosystems via scRNA‐seq. CA tumors displayed high *EPCAM*, *MUC5B*, *KRT8*, *KRT18*, and *AQP3* expressions, linked to cell cycle and metabolism. SCC tumors expressed *KRT6A*, *TP63*, *CDKN2A*, *KRT13*, and *DSG3*, related to hypoxia, EMT, and inflammation. This revealed tumor heterogeneity and distinct transcriptional patterns. Compared to SCC, CA has weaker immune infiltration, inactive TME, and higher cytokine/chemokine expression.

Lin et al. [38] compared SCC and CA TMEs and drug responses via single nuclei RNA sequencing (snRNA‐seq). Through investigating the heterogeneity of the malignant epithelial cell transcriptome, the research has identified specific chemotherapy drugs targeting SCC (dasatinib and doramapimod) and CA (pyrimethamine and lapatinib). The cell‐cell communication networks revealed that the NRG1‐ERBB2 and FN1‐ITAG3 pathways are specific to CA and SCC respectively, which may partially explain the specificity of the discovered chemotherapy drugs.

Peng et al. [40] investigated the intratumoral heterogeneity and immunosuppressive microenvironment of CA. Compared to SCC, CA exhibited a unique enrichment in several epithelial cell subclusters with elevated stemness and hyper malignancy characteristics, manifesting a highly immunosuppressive environment characterized by the enrichment of Tregs and tumor‐promoting neutrophils. Importantly, Solute carrier family 26, member 3 (SLC26A3) was identified as a promising diagnostic biomarker for predicting lymph node metastasis in patients with stage IIIC CA.

Qiu et al. [39] analyzed SCC, HPV‐associated CA, and HPV‐independent CA using both scRNA‐seq and T‐cell receptor sequencing (TCR‐seq). The study successfully established organoid models, validating the importance of endothelial cells and cancer‐associated fibroblasts (CAFs) in cancer development and progression. Based on the investigation of key driver genes, differentially expressed genes, and immune checkpoints, the authors developed precisely targeted therapeutic strategies for tumor epithelial cells, non‐malignant epithelial cells, CAFs, and T cells.

Minimal deviation adenocarcinoma of cervix (MDA) is a rare subtype that is not dependent on HPV. Zhang et al. [41] compared it with HPV‐associated cervical cancer using scRNA‐seq and found different immune cell compositions, with fewer B cells but a higher abundance of dendritic cells, monocytes, and T cells.

In summary, compared to SCC, AC exhibits many unique characteristics in both malignant tumor cells and TME composition. For instance, AC tumor cells have a higher degree of malignancy, and their immune microenvironment is more immunosuppressive. Therefore, future research should focus on the underlying mechanisms of AC based on these characteristics and explore potential treatment strategies.

## 6 Preclinical Research Models

To understand the underlying mechanism for cancer progression, appropriate preclinical models that can capture cancer's core characteristics are crucial [42]. Here, we summarize the common models used in CA research, including cell lines, animal models, and organoids, and discuss their respective advantages and disadvantages.

Cell lines, grown in vitro, can undergo repeated freezing‐thawing and divide indefinitely while maintaining their original characteristics. They're valuable for studying cancer biology and evaluating treatments [43]. However, commercially available CA cell lines are limited. In a study of 52 cervical cancer cell lines from 1960 to 2020, only 25 (48%) were from cervical tissues, and the remaining lines (52%) lacked proper identification [44]. Among the 25 cell lines, 19 (76%) were described as epithelial carcinomas, 3 (12%) were designated as adenocarcinomas (×H1, SiSo, RSBS‐43), 2 (8%) were unspecified as either squamous or adenocarcinomas (CA and SKG‐III), and 1 (4%) was stated to originate from histologically confirmed lymphoepithelioma‐like carcinoma (HUUCLE). This indicates a lack of sufficient CA cell lines to represent the diverse genetic backgrounds of CA.

Although maintaining cell lines is easy and cost‐effective, they have several limitations in research, such as lacking tumor heterogeneity and cell‐TME interactions [45]. To overcome these limitations, researchers often use experimental animals [46, 47, 48], organoids [49, 50, 51], and patient‐derived tumor xenografts (PDX) et al., as these models provide a closer approximation to tumor development. Organoids have been used in various cancers, including gynecological tumors [52]. Lee et al. [53] generated 15 cervical cancer‐derived organoids from 30 cancer tissues, including 60% SCC, 33.3% CA, and 6.7% neuroendocrine carcinomas. Liu et al. [54] established the largest cervical cancer PDX biobank to summarize treatment responses and evaluate new therapies. Hiroshima et al. [55] established a HER‐2+ SCC PDX model. Additionally, genetically engineered mouse models such as *K14E6* and *K14E7* were powerful tools for cervical cancer research [56, 57, 58]. However, establishing cervical cancer organoid models is still challenging, with low success rates of 50% for SCC and 25% for CA due to limited cell material from tumors [59]. The PDX model retains the TME and cellular characteristics of the primary tumor, yet it is expensive and the overall success rate of cervical cancer PDX models stands at 62.2% [60]. What's more, the existing studies have only reported on the SCC PDX and genetically engineered mouse model, and no CA PDX model has been discovered at the moment.

In sum, there is a scarcity of preclinical models for cervical cancer, particularly in CA. Consequently, the development of suitable and specific models in the future will be crucial for breakthroughs in diagnosis and treatment.

## 7 Summary and Prospects

HPV primarily infects stratified squamous epithelial cells of the skin and mucous membranes and replicates and proliferates in these cells. It has been suggested that epithelia with a glandular component may provide a microenvironment that favors HPV replication. This microenvironment may contain specific cytokines, growth factors, extracellular matrix components, and complex cell‐to‐cell interactions, which together act on HPV‐infected cells and may influence viral replication efficiency and pathogenicity [61, 62]. However, further scientific studies are needed to reveal the exact mechanisms of how epithelia with glandular components specifically affect HPV replication. With the increasing popularity of cervical cancer screening and the use of HPV vaccines, the incidence of SCC has significantly declined. In contrast, the relative incidence of CA has risen, displaying a trend of affecting younger individuals [1].

When it comes to diagnosis, CA poses greater challenges compared to SCC. The introduction of the Silva classification has provided a new perspective for the diagnosis of invasive CA, contributing to the avoidance of overtreatment. However, Silva classification is currently limited to conventional CA and has not yet encompassed other special histological subtypes of invasive CA [21]. Additionally, Silva classification's accuracy heavily relies on pathologists' diagnostic abilities, and studies have found that the consistency of pathologists' judgments across different subtypes is not entirely uniform, with the lowest consistency observed in Silva B diagnosis [21, 63]. Therefore, there is an urgent need to explore novel and reliable diagnostic markers to improve the accuracy of CA diagnosis.

As mentioned above the TME plays an important role in the development of cervical cancer and is heterogeneous across pathology types. Immunotherapy offers new hope for clinical treatment. Antibodies like cadonilimab [64] and zimberelimab [65] have demonstrated antitumor activity and safety in cervical cancer. Atezolizumab combined with bevacizumab and platinum improves survival [66]. Durvalumab with chemoradiotherapy is tolerated well in locally advanced cervical cancer [67]. However, due to the relatively inactive immune infiltration in CA, it is insensitive to immunotherapy. Currently, there is a lack of large‐scale clinical studies on immunotherapy specifically targeting CA. Future clinical research is needed to identify better biomarkers that can define CA patients who are likely to benefit from immunotherapy.

Currently, CA research lacks sufficient preclinical models. Organoids can closely mimic the structure and function of tumor tissue in patients, providing precise and individualized in vitro models for drug efficacy prediction and disease modeling. Therefore, future efforts should be directed towards developing individualized in vitro models, such as organoids et al., specific to different pathological subtypes, to better guide clinical treatment and research. Furthermore, the development of genetically altered mice based on the genetic profiles of various histological subtypes is an area that requires further investigation.

Altogether, our current understanding of CA remains limited, future studies should concentrate on the development of more sophisticated preclinical models, evaluation of the efficacy of immunotherapeutic interventions in CA patients, and exploration of reliable biomarkers via comprehensive multi‐omics analysis.

## Author Contributions

**Shuhui Li:** writing – original draft (equal). **Congrong Liu:** writing – review and editing (equal). **Liang Weng:** writing – review and editing (equal).

## Ethics Statement

Authors must declare all information about ethics in this section including following as appropriate: Approval of the research protocol by an Institutional Reviewer Board: N/A; informed consent: N/A; Registry and the Registration No. of the study/trial: N/A; animal studies: N/A.

## Conflicts of Interest

The authors declare no conflicts of interest.

## Data Availability

The authors have nothing to report.
